# Growth Hormone Replacement Therapy: Transition from Adolescence to Adulthood

**DOI:** 10.4274/jcrpe.v1i5.205

**Published:** 2009-08-01

**Authors:** Mitchell E. Geffner

**Affiliations:** 1 Saban Research Institute, Childrens Hospital Los Angeles, Keck School of Medicine, University of Southern California, Los Angeles, USA; +00 323−361−7032 +00 323−361−1350mgeffner@chla.usc.eduChildrens Hospital Los Angeles, 4650 Sunset Blvd., Los Angeles, CA 90027, USA

**Keywords:** growth hormone, transition, adolescence, metabolic

## Abstract

Consideration of GH re−testing should be performed in all adolescents reaching the transition period (if not at start of puberty) who had been previously diagnosed with idiopathic, isolated GH deficiency. In the presence of multiple hormone deficiencies and/or clear−cut evidence of organic disease, persistence of severe GH deficiency is much more likely. Thus, GH deficiency may be “confirmed” by a low serum IGF−I concentration. During the transition period, the optimal time to reassess the integrity of the GH−IGF−I axis after prior GH treatment, the specific testing protocol to use, and the definition of GH deficiency all remain unknown. During the transition period, patients should have their GH dose lowered with (upward) adjustments made on the basis of age−and gender−adjusted serum IGF−I concentrations. GH treatment during the transition period has been shown in most, but not all, studies to be beneficial in preventing development of the features of the adult GH deficiency syndrome. It is important to remember that, during the transition period in teenagers with GH deficiency, there must be initiation of a careful plan for transfer of care to an intermist−endocrinologist with expertise in management of hypothalamic−pituitary disease in young adults.

**Conflict of interest:**None declared.

## INTRODUCTION

Until approximately 15 years ago, growth hormone (GH) treatment was confined mostly to children with GH deficiency (GHD). In 1997, GH treatment was first approved in the United States (US) for use in adults with GHD, either acquired in childhood or de novo in adults with hypothalamic−pituitary disease. Most recently, there has been increasing study of children previously diagnosed with GHD who have reached older adolescent age in terms of persistence of the original diagnosis, optimization of GH retesting and dosing, and evidence underscoring need for persistence of GH treatment. As such, this period of time between childhood and adulthood has been dubbed the “transition period” which refers to a broad set of physical and psychosocial changes, which are arbitrarily defined as starting in puberty and ending with full adult maturation. As it relates to GHD, this period is defined as the window of time in which a child no longer requires GH for sustaining linear growth, but does so for ongoing treatment of non−statural, i.e., metabolic, reasons. This usually suggests a period from the mid−to−late teenage years until 6−7 years after reaching adult height.

## ADULT GH DEFICIENCY SYNDROME

The need for GH treatment of GH−deficient adults is based on two general pieces of evidence. First, untreated adults with proven severe GHD manifest what has come to be known as the adult GHD syndrome. It is characterized by: (i) abnormal body composition (with increased fat mass and decreased lean body or muscle mass); (ii) decreased bone mineral density (with increased fracture risk possibly exacerbated by sex steroid deficiency and/or over−treatment with glucocorticoids); (iii) reduced exercise capacity (perhaps as a result of the aforementioned unfavorable body composition); (iv) abnormal serum lipids (including increased total and LDL cholesterol, increased triglycerides, and decreased HDL cholesterol); and (v) impaired quality−of−life (including social isolation, depression, and diminished sense of well−being) as assessed by standardized questionnaire. These findings (collated from 9 studies) are summarized in the Table 1, which shows the effects of severe GHD in young adults in whom GH treatment had been discontinued for an average of 8 years after attainment of final height (1, 2, 3, 4, 5, 6, 7, 8, 9). 

Second, the improvement of the above constellation of abnormalities with GH treatment (at relatively low doses compared to those required for statural growth in childhood) in double−blinded, placebo−controlled studies cemented the existence of the adult GHD syndrome.

**Table 1:**
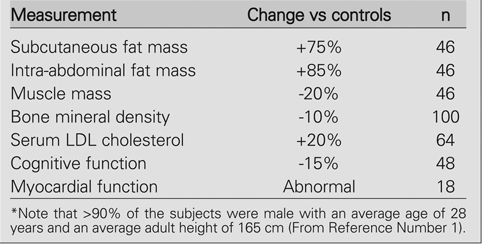
Effects of Severe GH Deficiency in Young Adults*

## PERSISTENCE OF CHILDHOOD GH DEFICIENCY

With the approval of GH treatment in the US by the FDA to treat adult GHD, studies commenced to assess the magnitude of newly diagnosed GHD or re−assess the severity of previously diagnosed disease. Surprisingly (at first), the “pass rate” (i.e., no evidence of GHD) on re−testing of older adolescents previously diagnosed with childhood GHD was strikingly high. In those previously diagnosed as having had idiopathic, isolated GHD, as many as 67% of subjects now passed their re−test, i.e., had peak GH values >10 mg/L following stimulation. In fact, similar results have recently been reported when retesting was done at the start of puberty (10). However, in the presence of multiple hormone deficiencies and/or clear−cut evidence of organic hypothalamic−pituitary disease, persistence of severe GHD into adulthood was >90% (11). While an entity of transient GHD in childhood might exist, this difference in results could more likely reflect variations in stimulation testing protocols, reproducibility of GH test results, methodological factors in GH assays, failure to use sex−steroid priming prior to provocation, and concomitant effects of nutrition.

## GH RE-TESTING: WHOM?

Thus, re−evaluation of the GH−IGF−I axis should be performed in all adolescents reaching the transition period (if not at start of puberty) who had been previously diagnosed with idiopathic (normal head MRI), isolated (no other hypothalamic−pituitary hormone deficiencies) GH deficiency. Patients with a high likelihood of having permanent GHD are those who have multiple pituitary hormone deficiencies (MPHD) and a low serum IGF−I concentration in conjunction with one or more of the following: (i) a clinically and/or radiologically confirmed congenital mid−line abnormality involving the sellar or suprasellar region; (ii) known acquired hypothalamic−pituitary disease, e.g. due to a craniopharyngioma; (iii) prior surgery for lesions directly affecting the hypothalamic−pituitary region or high−dose radiotherapy for malignant disease located in the hypothalamic−pituitary region; or (iv) a proven genetic/molecular defect involving the proximal portion of the GH−IGF−I axis. If children in these high−risk categories have a low serum IGF−I level after short−term discontinuation of GH treatment, this should suffice as documentation of persistent GHD (12).

## GH RETESTING: WHEN?

Since longstanding GH treatment may well blunt the response to acute GH stimulation testing by having caused a chronic increase in the serum level of IGF−I, a GH−free “wash−out” period is recommended prior to retesting. However, the shortest interval off GH therapy that would allow valid re−testing of the GH axis remains unknown. Proposed intervals between cessation of GH treatment and re−testing of the GH axis generally range from 1 to 3 months (13). Optimization of all other hormone therapy doses is critical for accurate interpretation of GH testing, especially thyroid hormone.

## GH RETESTING: HOW?

**Figure 1:**
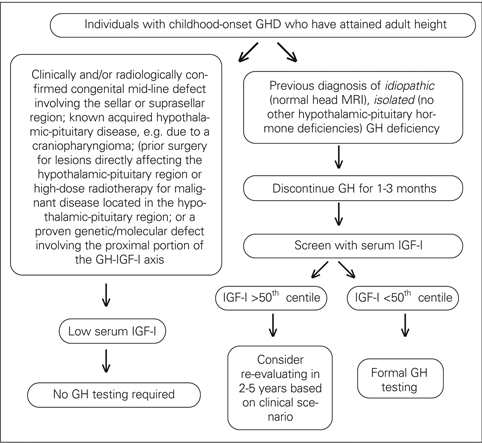
GHD: Schema for reassessing the GH−IGF−I axis during the transition period

This process should start with measurement of a serum IGF−I concentration which, if ≥50^th^ %ile for age and gender, effectively rules out persistent GHD which is the expected outcome in those with idiopathic, isolated GHD. If the IGF−I level is ≤50^th^% ile in individuals with a high likelihood of persistent GHD (see “GH Testing: Whom”), formal GH testing need not be done, but, in those with a presumed low likelihood, i.e., those with a prior diagnosis of idiopathic, isolated GHD, formal retesting is mandatory (13) (Figure 1).

In childhood, GHD in the US has traditionally been defined as all stimulated GH values <10 mg/L using traditional stimuli such as clonidine, carbidopa, glucagon, arginine, and/or insulin. In adults, insulin hypoglycemia is the test of choice to diagnose GH deficiency, with severe deficiency arbitrarily defined as a peak GH of <3 mg/L. However, as this test is labor−intensive, expensive, and not without risk, internist−endocrinologists developed alternative combination protocols including GH−releasing hormone (GHRH)/arginine (preferred in the US) and GHRH/pyridostigmine (used in Europe). Because of the potency of these combination stimuli relative to insulin, severe GHD was defined as a peak GH of <9 mg/L. However, GHRH is no longer available in the US, so additional testing protocols for adults need to be devised (14).

For diagnosis of GHD during the transition period, the proposed cut−off is a GH level of <5 mg/L, intermediate between the criteria for childhood and adult GHD. Unfortunately, during this period, the optimal means of GH retesting is unknown. Although not well studied in this age group, the preferred tests are insulin or glucagon.

In ultimately deciding what is the appropriate GH cut−off to diagnose GHD during the transition period, it is important to consider the normal gender−, age− and/or pubertal−adjusted pattern of GH secretion (recognizing that the greatest amount of GH is produced during puberty). The effect of BMI (e.g., as reflected by waist−to−hip ratio) must also be taken into account as GH secretion (at all ages) decreases with increasing visceral adiposity (15).

## GH TREATMENT: WHY?

The bulk of existing data reinforce the need for GH treatment of adults with persistent GHD previously diagnosed in childhood. Supporting this recommendation are several key studies. First, in a 2−year study of GH resumption (comparing two doses, 12.5 and 25 μg/kg/day vs. placebo) during the transition period among 64 individuals previously treated for childhood GHD and off GH for at least 1 year, bone mineral density of the spine was shown to increase significantly in both treatment arms after 2 years of treatment with no significant improvement in the placebo group (16).

Second, 149 older adolescents (mean age 19 years) with severe GHD (80% with MPHD) were randomized to receive one of two different GH dosages or placebo for 2 years (17). In both treatment arms, significant favorable differences in body composition were found, i.e., increased lean body mass and decreased fat mass, with absence of disturbances in LDL and HDL concentrations. These investigators concluded that, when two−dimensional statural growth is complete, children with GHD still need GH to achieve three−dimensional development to target adult body composition, and that a long period off GH can be detrimental.

Third, using combined KIGS (Pfizer International Growth)−KIMS (Pfizer International Metabolic) pharmaco−epidemiological databases (18), data from 324 patients (200 males), initially followed in KIGS and then re−enrolled into KIMS, with a mean duration between the end of pediatric and the resumption of adult GH treatment of 4.5±3.1 yr, were analyzed. The results showed that serum IGF−I SDS in adults was related to the severity of GHD during childhood and that a longer period between cessation of GH treatment at the completion of linear growth and recommencement of GH treatment was associated with statistically significant detrimental lipid profiles and poorer quality−of−life. The investigators concluded that pediatricians should tailor GH treatment not only for its effect on childhood GHD, but also for future health in adulthood. 

Fourth, in another study of 58 adolescents with childhood−onset GHD, a large proportion of whom had isolated idiopathic GHD, investigators found contrasting observations (19). These individuals were randomized to receive either GH or placebo for 2 years after retesting at study start confirmed the diagnosis of GHD. After 2 years, no difference existed between the two groups with regard to lipid and carbohydrate metabolism, body composition, bone mineral density, cardiac function, muscle strength, or quality−of−life. Thus, these investigators concluded that GH can safely be discontinued for at least 2 years after attainment of near−adult height.

## GH DOSING DURING TRANSITION: HOW MUCH?

During puberty, adolescents with GHD typically receive GH in a wide range of doses (usually between 1.25 and 2.5 mg/day for a child weighing 50 kg, although the FDA has approved doses as high as 5 mg/day in the US). In the transition period, patients should have their GH dose lowered or, if already discontinued, restarted between 0.2−0.5 mg/day with (upward) adjustments made on the basis of age− and gender−adjusted serum IGF−I levels, estrogen status, clinical response, and lack of side effects (12).

## SUMMARY AND CONCLUSIONS

A schema by which to begin the re−evaluation process for adolescents/young adults previously diagnosed with childhood−onset GHD can be found in the Figure and can serve as the basis for transitioning the pediatric patient to adult care.

Somatic and biochemical targets during the transition period are bone mineral accretion and serum IGF−I concentrations. In patients with idiopathic, isolated GHD, GH re−testing should be performed. In patients with MPHD and a low serum IGF−I concentration, there is generally no need to re−test GH secretory status and GH should be continued at a lower dose than previously used for height achievement. Even with confirmed severe GHD, if the serum IGF−I concentration and bone density are normal, there appears to be no negative impact of treatment discontinuation for 2 years.

While the benefits and risks of GH treatment in adults have been extensively studied over the past 15 years, criteria for who should be tested, optimal method(s) of testing, and time off GH prior to retesting of GH axis in young adults with childhood−onset GHD during the transition period remain much less well−characterized. Retesting of other hypothalamic−pituitary axes for which treatment had or had not been initiated in childhood has received even less attention, but possibilities of spontaneous normalization (e.g., thyroid function) or late drop−out (e.g., adrenal function) must be considered. Finally, it is important to remember that, during the transition period in teenagers with GHD or more extensive hypopituitarism, there must be initiation of a thoughtful and seamless plan for transfer of care to an internist−endocrinologist with expertise in management of hypothalamic−pituitary disease in young adults (20).
